# Stereotactic body radiation therapy for renal cell carcinoma: a small number of initial clinical experiences

**DOI:** 10.1093/jrr/rraf028

**Published:** 2025-06-03

**Authors:** Takahiro Aoyama, Yutaro Koide, Hidetoshi Shimizu, Tomoki Kitagawa, Tohru Iwata, Shingo Hashimoto, Hiroyuki Tachibana, Takeshi Kodaira

**Affiliations:** Department of Radiation Oncology, Aichi Cancer Center, 1-1 Kanokoden, Chikusa-Ku, Nagoya, Aichi 464-8681, Japan; Department of Radiation Oncology, Aichi Cancer Center, 1-1 Kanokoden, Chikusa-Ku, Nagoya, Aichi 464-8681, Japan; Department of Radiation Oncology, Aichi Cancer Center, 1-1 Kanokoden, Chikusa-Ku, Nagoya, Aichi 464-8681, Japan; Department of Radiation Oncology, Aichi Cancer Center, 1-1 Kanokoden, Chikusa-Ku, Nagoya, Aichi 464-8681, Japan; Department of Radiation Oncology, Aichi Cancer Center, 1-1 Kanokoden, Chikusa-Ku, Nagoya, Aichi 464-8681, Japan; Department of Radiation Oncology, Aichi Cancer Center, 1-1 Kanokoden, Chikusa-Ku, Nagoya, Aichi 464-8681, Japan; Department of Radiation Oncology, Aichi Cancer Center, 1-1 Kanokoden, Chikusa-Ku, Nagoya, Aichi 464-8681, Japan; Department of Radiation Oncology, Aichi Cancer Center, 1-1 Kanokoden, Chikusa-Ku, Nagoya, Aichi 464-8681, Japan

**Keywords:** renal cell carcinoma, stereotactic body radiation therapy, dosimetric analysis, initial clinical experience

## Abstract

Stereotactic body radiation therapy (SBRT) has emerged as a promising and minimally invasive treatment option for patients with renal cell carcinoma (RCC). This study presents our initial clinical experiences with treatments following our center’s protocol, which was formulated based on both national and international evidence. Six patients who had undergone renal SBRT at our center from January 2021 to December 2023 were included. Treatment planning used computed tomography (CT) and magnetic resonance imaging, with respiratory management conducted through breath-hold or free-breathing techniques. The prescribed dose was primarily 48 Gy in three fractions, with increased fractionations when dose constraints were challenging to achieve. Dose constraints were met for all patients, and treatment planning adhered to protocol guidelines. After the confirmation of cone-beam CT (CBCT) images by physicians, radiation was delivered. Five out of six patients completed the planned treatment, whereas one discontinued the treatment midway (the causal relationship to radiation therapy was unclear). Dose–volume histogram analysis revealed that doses to organs at risk depended on the position and size of the planning target volume but remained within acceptable limits for all cases. The intrafractional patient motion was 2.7 mm, as calculated from the pre- and post-CBCT images, confirming the appropriateness of a 3-mm setup margin. Although this study provides initial insights, further clinical trials are warranted to establish standardized protocols and optimize treatment strategies for RCC. In the future, it is also necessary to generate evidence that is tailored to the current situation in Japan.

## INTRODUCTION

Stereotactic body radiation therapy (SBRT) offers a potent and minimally invasive treatment option for patients with renal cell carcinoma (RCC) who are medically inoperable, at high surgical risk, or refuse to undergo surgery. Moreover, it has shown promise in controlling local disease and preserving renal function [1, 2]. The nonrandomized phase II trial administered SBRT to patients with inoperable or high surgical risk tumors, achieving 100% local control at 12 months [3]. It reported few grade 3 adverse events and no cancer-related deaths. These promising results suggest that SBRT can act as a key component in RCC treatment strategies [4].

In Japan, the 2018 revision of medical service fees expanded the use of SBRT to include renal cancer. Advances in treatment equipment are enhancing accuracy, raising expectations for SBRT to become widely adopted, akin to its use in lung and liver cancer therapies, which were covered by insurance earlier. However, a 2020 national survey reported that only 0.3% (21/7079 cases) of the annual renal cancer cases involved SBRT [5]. This low rate may be attributed to the lack of comprehensive SBRT protocols in existing national and international guidelines [6, 7]. In addition, uncertainties remain regarding respiratory management methods, dose constraints for normal organs, and established radiotherapy procedures. The limited number of cases has also slowed proficiency, hindering SBRT’s widespread use.

This study reports our initial clinical experiences with treatments following our center’s protocol, which was developed based on both national and international evidence. Through dosimetric analyses, we identified issues encountered during the initial experience and outlined necessary updates, thereby contributing to the broad adoption of SBRT for RCC treatment in Japan.

## MATERIALS AND METHODS

### Patient selection

This retrospective study included all patients who received SBRT targeting the kidneys between January 2021 and December 2023. Our study protocol was approved by the Institutional Ethics Review Committee (2024-0-489). All assessments were conducted in accordance with the relevant guidelines and regulations. All patients underwent computed tomography (CT) every 2–3 months to evaluate tumor response, and toxicity was evaluated according to Common Terminology Criteria for Adverse Events version 4.03.

### Treatment planning CT and contouring

Treatment planning CT was performed with 2-mm slices. All patients were immobilized using vacuum cushions (Vac-Lok, CIVCO Medical Systems, CA, USA, or BlueBag, Elekta, Stockholm, Sweden) with their arms positioned down to ensure reproducibility of positioning. For respiratory management, breath-hold irradiation was the preferred choice for patients able to hold their breath using the Abches system (Apex Medical, Tokyo, Japan). The CT protocol comprised plain scans for treatment planning and dynamic three-phase contrast-enhanced scans. For patients unable to maintain breath-hold, free-breathing irradiation was the preferred approach, employing chest–abdominal compression (Bodyfix, Elekta, Stockholm, Sweden), and four-dimensional (4D) CT was performed.

The gross tumor volume (GTV), which is equivalent to the clinical target volume (CTV), was delineated on all CT images acquired during treatment planning, and the CTVs were summed to obtain an internal clinical target volume [iCTV = internal target volume (ITV)]. A 3-mm setup margin (SM) was added to the ITV to define the planning target volume (PTV).

### Prescription dose and optimization calculation

Dose calculation was performed using RayStation version 10.0 (RaySearch Laboratories, Stockholm, Sweden) with the collapsed cone convolution algorithm or Pinnacle3 version 9.10 (Philips Radiation Oncology Systems, Fitchburg, WI, USA) with adaptive convolution algorithm. The prescribed dose was 48 Gy in three fractions (D_95_: 95% of the PTV volume receives the prescribed dose), and the maximum dose within the PTV was set to 125%–130% of the prescribed dose [8]. The dose constraints for organs at risk, based on previous studies [9, 10], are presented in Table 1. If these dose constraints could not be met, the prescription dose and fractionation were modified to D_95_ = 70 Gy and 10 fractions, respectively. If the dose constraints remained difficult to achieve, a gradual reduction to D_95_ = 60 or 50 Gy in 10 fractions was allowed. Respiratory motion was evaluated using treatment planning CT. Patients with tumor motion ≥1 cm owing to respiration underwent seven-field noncoplanar three-dimensional irradiation to minimize the impact of the interplay effect among the multi-leaf collimator, jaw, gantry movements, and target motion [11]. For those with tumor motion <1 cm, volumetric modulated arc therapy (VMAT) with a half arc was used. Before initiating treatment, all treatment plans were approved by two radiation oncologists and confirmed using a semiconductor dose verification device (MapCHECK2, Sun Nuclear, Melbourne, FL, USA) according to the guidelines [12].

**Table 1:** Dose constraints for stereotactic body radiation therapy for renal cell carcinoma

**Organ at risk**	**Metric**	**Fraction number**	
		**3 fractions**	**10 fractions**
Spinal cord	D_0.03 cc_	18 Gy	
	D_max_	22.2 Gy	35 Gy
Small bowel	D_10 cc_	11.4 Gy	43 Gy
	D_1 cc_	24 Gy	52 Gy
	D_max_	30 Gy	
Stomach	D_10 cc_	16.5 Gy	43 Gy
	D_5 cc_	22.5 Gy	
	D_1 cc_		52 Gy
	D_max_	30 Gy	
Duodenum	D_10 cc_	11.4 Gy	43 Gy
	D_5 cc_	16.5 Gy	
	D_1 cc_	22.2 Gy	52 Gy
	D_0.5 cc_		
Large bowel	D_10 cc_		43 Gy
	D_1 cc_		52 Gy
	D_max_	30 Gy	
Skin (5 mm inside from external)	D_10 cc_	30 Gy	58 Gy
	D_1 cc_		71 Gy
	D_0.5 cc_^a^	33 Gy	
Liver	D_700 cc_	15 Gy	
	V_17 Gy_	66%	
	D_10 cc_		58 Gy
	D_1 cc_		71 Gy
Contralateral kidney	V_10 Gy_	33%	
	V_5%_	14Gy	
	Mean		30 Gy
Great vessels	D_10 cc_	39 Gy	58 Gy
	D_1 cc_		71 Gy
	D_0.03 cc_	45 Gy	
Ureter	D_10 cc_		58 Gy
	D_1 cc_		71 Gy
	D_0.03 cc_	40 Gy	

^a^The D0.5 cc skin constraint was added after Patient 3.

### Treatment delivery

Patients were treated using linear accelerators (TrueBeam, Varian Medical Systems, Inc., Palo Alto, CA, or Synergy, Elekta, Stockholm, Sweden). After bone structure matching using cone-beam CT (CBCT) images and the physicians’ confirmation that the tumor was located within the ITV, radiation was delivered. The respiratory phase during irradiation was the same as that established during treatment planning (breath-hold or free-breathing irradiation), and respiratory management during irradiation (e.g. synchronized irradiation) was not performed. After beam irradiation, CBCT was performed again to calculate the amount of intrafractional patient motion [13].

## RESULTS

### Patients

Six patients underwent renal SBRT during the study period. Table 2 presents detailed patient characteristics. As Patient 2 could not maintain an adequate breath-hold, treatment was delivered under free-breathing conditions. In addition, Patients 2 and 6 required a shift from 3 to 10 fractions owing to difficulties in meeting the dose constraints. A median follow-up period of 12 (range, 6–23) months was observed. Among the six patients, three died from causes unrelated to RCC during the follow-up period, one was lost to follow-up after being transferred to another institution, and two survived at the time of data analysis. Regarding the treatment site, only one patient (Patient 2) experienced local disease progression at 12 months, with no other patients demonstrating progression during the follow-up period.

**Table 2:** Patient and treatment characteristics

**Patient No.**	**1**	**2**	**3**	**4**	**5**	**6**
Age, sex	74, M	76, M	49, M	80, M	57, M	71, M
PS	0	1	0	0	0	0
Days from planning CT	19	15	14	13	20	17
Primary tumor category	1a	1a	3a	1a	1b	1a
Tumor site	R	R	R	L	L	L
GTV (ml)	1.92	40	200.1	19.8	43.7	36.4
PTV (ml)	7.37	67.8	262.5	44.3	86.8	61.8
Respiratory motion^a^ [mm]	3.9	14.0	3.0	2.8	6.2	2.4
Fraction	3	10	3	3	3	10
Prescription dose (Gy)	48	50	48	48	48	50
Monitor unit	3613	935	3225	4578	4160	1315
Technique	VMAT	7 field	VMAT	VMAT	VMAT	VMAT
Beam on time (s)	91	112	82	130	109	39
Median treatment time [min] (range)	53	57	52	38	64	41
	(45–76)	(51–61)	(38–53)	(32–45)	(55–67)	(26–50)

Abbreviations: PS, performance status; CCI, Charlson comorbidity index; CT, computed tomography; GTV, gross tumor volume; PTV, planning target volume; VMAT; volumetric modulated arc therapy.

^a^Respiratory motion refers to the reproducibility of breath-hold in patients 1, 3, 4, 5, and 6, and tumor motion due to respiration in patient 2.

### Treatment planning and delivery

**Fig. 1:**
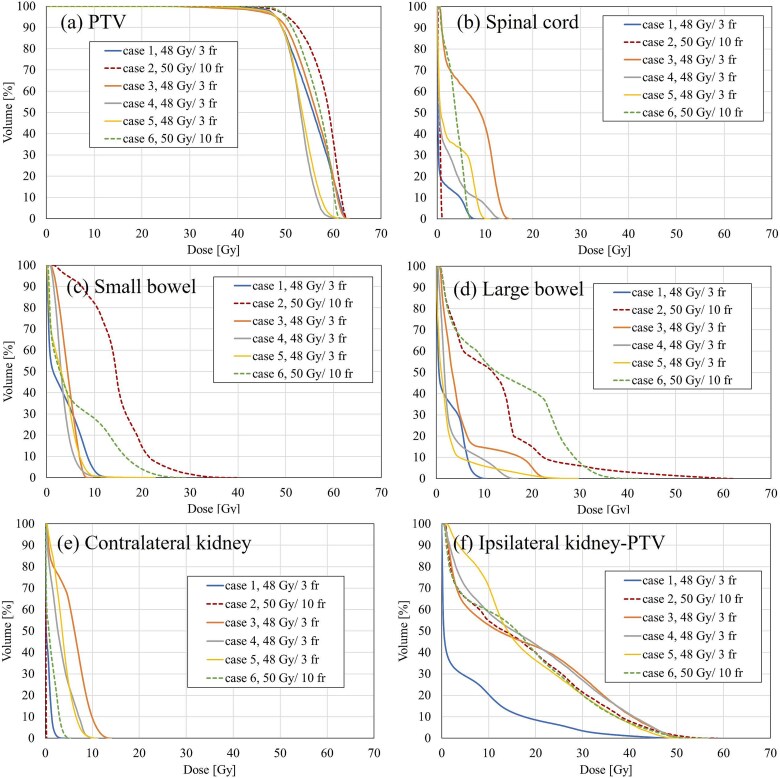
Dose–volume histogram for each patient’s (a) planning target volume (PTV) and (b–f) organs at risk.

Dose–volume histogram (DVH) analysis results for all patients are presented in Fig. 1. All patients met the dose constraints outlined in Table 1; however, two patients treated with 10 fractions (Patients 2 and 6) received a dose of 5 Gy per fraction owing to intestinal dose constraints. Patient verification showed adequate quality assurance results in all cases. The intrafractional patient motion was 2.7 mm, as calculated from the pre- and post-CBCT images.

### Key cases

**Fig. 2:**
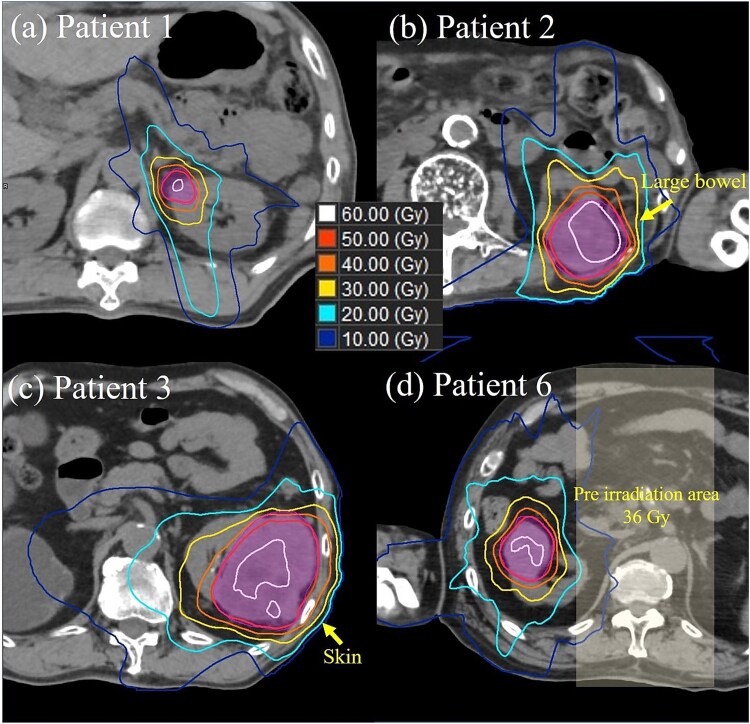
Axial dose distribution in key cases. (a) Patient 1, who was the first patient at our center, treated with 48 Gy in three fractions under breath-hold conditions. Filled contours indicate the planning target volume (PTV). (b) Patient 2, for whom 10 fractions were selected owing to large bowel (yellow arrow) dose constraints. (c) Patient 3, with the largest PTV volume (262.5 ml) among all patients; scarring was observed on the skin (yellow arrow) 6 months after irradiation. Patient 6, who had a history of abdominal irradiation (36 Gy) and for whom 10 fractions were selected to manage accumulated dose constraints.

*Patient 1 (* *Fig. 2a* *)*

Patient 1 was the first case at our center. The tumor, with a maximum diameter of 8 mm, was located on the upper inner side of the right kidney. Treatment was delivered under breath-hold conditions with a dose of 48 Gy in three fractions. The monitor unit value was 3613, with a beam-on time of 91 s. Time from entry-to-exit was the longest on the first day (76 min) and decreased on subsequent days (45 and 53 min).

*Patient 2 (* *Fig. 2b* *)*

Patient 2 had a medical history of malignant myeloma and a spinal compression fracture. Due to breath-holding difficulty, free breathing with chest–abdominal compression was selected. The respiratory motion was 1.4 cm, confirmed via 4D-CT, using a noncoplanar seven-field beam. Given the proximity between the tumor and the bowel, achieving the 3-fraction constraint was unfeasible; therefore, 10 fractions were used. Even with 10 fractions, 5 Gy per fraction was chosen owing to large bowel dose limitations. After 7 of the planned 10 fractions, treatment could not be continued and irradiation was ceased after the 8th fraction.

*Patient 3 (* *Fig. 2c* *)*

Patient 3 exhibited the largest tumor volume (GTV = 200.1 ml) among all cases. Treatment involved 48 Gy in three fractions, with treatment planning and delivery adhering to the protocol. The skin dose was D_10 cc_ = 27.6 Gy, meeting the dose constraint (<30 Gy). However, 6 months post treatment, scarring was observed near the irradiated area.

*Patient 6 (* *Fig. 2d* *)*

Although the tumor volume was moderate at 36.4 ml, Patient 6 had a history of prior irradiation (36 Gy in 20 fractions for malignant lymphoma) in the same area. Considering the cumulative dose with biologically effective dose (BED; α/β = 3) from the previous and current irradiations, 50 Gy in 10 fractions was selected owing to constraints in the gastrointestinal tract dose.

## DISCUSSION

We herein present our clinical experience with SBRT for RCC. Protocol-based treatment planning and delivery was performed in all but one patient. Considering the limited number of annual cases undergoing renal SBRT in Japan [5], reporting initial experiences, as in this study, is tremendously valuable. Although the number of patients included in this study was insufficient for a meaningful comparison of outcomes based on radiation therapy dose fractionation, none of the patients who received the planned dose experienced local progression.

DVH analysis confirmed that dose constraints were met, although the organs-at-risk dose depended on the location and size of the PTV. In total, 2/6 patients could not meet the 3-fraction constraint and were switched to 10 fractions. Five of the six patients completed the treatment as planned. However, one (Patient 2) was terminated midway. During treatment, the patient experienced gastrointestinal symptoms (grade 2), general malaise (grade 3), and worsening back pain, likely related to comorbidities, which led to treatment discontinuation. The causal link to radiotherapy was unclear. Future considerations should weigh the prescribed dose and fractionation against safety.

The intrafractional patient motion, calculated from the pre- and post-irradiation CBCT images, was 2.7 mm, consistent with a previous study [14]. Considering the accuracy of device operation and interoperator variability, the 3-mm SM in our protocol was confirmed to be appropriate [15]. However, it should be noted that this study was unable to observe changes in the amount of respiratory movement or patient motion during irradiation. In VMAT cases, the average beam-on time was 90.2 seconds, allowing treatment within 5–6 breath holds, and the median entry-to-exit time was 45 minutes, similar to other SBRT [16]. Non-coplanar fixed irradiation did not require breath hold but had a longer median time of 57 minutes due to couch repositioning.

In Patient 3, despite meeting the skin dose constraint, skin scarring (grade 1) was observed 6 months after irradiation. A further literature review revealed the significance of D_0.5 cc_ dose constraints (<33 Gy), which were not included in our initial protocol [17]. For Patient 3, the D_0.5 cc_ dose was 37.4 Gy, exceeding the constraint. After replanning, we achieved the D_0.5 cc_ constraint (32.7 Gy) without compromising PTV coverage and added a D_0.5 cc_ < 33 Gy constraint for subsequent cases. Skin-related adverse events resulting from SBRT have been noted in a previous study [18]. This case was reported to our center’s quality control committee, and the information was shared with the radiation oncology staff.

This study had several limitations. First, it reports on initial experiences with a small number of patients at a single institution, and clinical outcomes were not extensively evaluated owing to the short follow-up period. Nevertheless, ensuring safe treatment is imperative, and clinical outcomes are currently being investigated. In addition, the kidneys are subject to respiratory motion, thereby necessitating active respiratory management in some cases. In particular, the current study did not evaluate the extent of respiratory movement in patients during treatment. Hence, future studies will need to evaluate the SM by implementing methods such as fluoroscopic monitoring during irradiation. To address these issues, large-scale randomized controlled trials are necessary. However, given the limited number of cases requiring SBRT for RCC in Japan, conducting such trials might prove challenging. Therefore, alternative approaches, such as nonrandomized parallel-group comparison studies using advanced statistical methods, could nonetheless provide valuable insights despite the limited patient cohort. These approaches can generate evidence appropriate for the current situation in Japan.

In conclusion, this study reported our initial clinical experiences with renal SBRT at our center, from treatment planning to protocol execution. Intrafractional patient displacement was 2.7 mm, thereby validating the 3-mm SM. Prescribed doses and dose constraints should continue to be refined with updates from the latest evidence. Although this study provides initial insights, further clinical trials are warranted to establish standardized protocols and optimize treatment strategies for RCC.
